# A qualitative study of mothers’ health literacy related to malnutrition in under 5-year-old children in southern Mozambique

**DOI:** 10.1017/S1368980021004365

**Published:** 2022-07

**Authors:** Leona Lindberg, Inocência Nhambongo, Tacilta Nhampossa, Khátia Munguambe, Gunilla Priebe

**Affiliations:** 1 School of Public Health and Community Medicine, Institute of Medicine, Sahlgrenska Academy, University of Gothenburg, Box 463, Goteborg, 40530, Sweden; 2 Social Science Unit, The Manhiça Health Research Centre, Manhiça, Mozambique; 3 Clinical Department, The Manhiça Health Research Centre, Manhiça, Mozambique

**Keywords:** Malnutrition, Mozambique, Health literacy

## Abstract

**Objective:**

To explore mothers’ perceptions of malnutrition and its causes in U-5’s in Mozambique, as well as their ability to recognise, prevent and act on signs of malnutrition.

**Design:**

A qualitative exploratory inquiry using focus group discussions and individual interviews analysed using Nutbeam’s health literacy themes.

**Setting:**

Manhiça District Hospital in Manhiça, Mozambique.

**Participants:**

Mothers of U-5’s (*n* 53) attending the in- and out-patient paediatric wards.

**Results:**

Different malnutrition literacy levels were identified in mothers’ responses. Mothers’ reflections on the causes of malnutrition in U-5’s were more elaborate compared to those of recognition, prevention and treatment strategies. Only severe forms of acute malnutrition were recognised by mothers, while early signs of undernutrition and stunting largely went undetected or unmentioned. Limited knowledge, time and financial resources were mentioned as contributors to suboptimal practices resulting in malnutrition. The district hospital, rather than community resources or local health posts, was indicated as the place mothers would go to seek advice and treatment for malnutrition. All mothers requested additional information on how to prevent and treat malnutrition.

**Conclusions:**

The varying literacy levels among mothers, the lack of references to community health workers as a resource in identifying and managing malnutrition, and the identification of poverty and sociocultural conditions as contributors to suboptimal practices indicate the need for in-depth research focused on the social determinants of malnutrition. A more comprehensive understanding of mothers’ health literacy would contribute to the development of holistic programmes aiming to improve community management of malnutrition.

Malnutrition plagues a large part of the Mozambican U-5 population, with <5 % wasted^(1)^, approximately 15 % underweight, >60 % anaemic and >40 % stunted (low height-for-age)^(2)^, putting it in the ‘very high’ bracket for stunting prevalence^(3)^. In the Government’s Multisectoral Action Plan for the Reduction of Chronic Undernutrition 2011–2015, the high prevalence of malnutrition in U-5’s has been attributed to the following factors: insufficient nutrient intake and illness from infections, household food insecurity, inadequate breast-feeding, pregnancy during adolescence, poor access to health and sanitation services, poverty, lack of access to education and gender inequality^(4)^. While these immediate, underlying and basic causes of malnutrition in U-5’s in Mozambique are well founded, mothers’ perceptions of the causes, effects and severity of malnutrition are, to our knowledge, not researched in this setting. Since studies from other contexts have shown that mothers’ perceptions of illnesses influence health-seeking behaviours^(5–9)^, and specifically, their conceptualisation of the aetiology of malnutrition and how this influences health-seeking behaviours or response to nutrition interventions^(5–7,9)^ gaining an understanding of how malnutrition is perceived by caregivers is necessary for efficient tailoring of nutrition intervention/rehabilitation programmes^(9–11)^.

With this rationale, this exploratory inquiry aimed to explore mothers’ perceptions of malnutrition and its causes in U-5’s in rural Mozambique, as well as their ability to recognise, prevent and act on signs of malnutrition, with the ultimate goal of identifying areas for further research related to mothers’ malnutrition literacy and health-seeking behaviours.

## Methods

### Study location

A qualitative exploratory inquiry, which employed focus group discussions (FGD) and individual interviews (IDI), was conducted at the Manhiça District Hospital (MDH), situated in the south of Mozambique. The MDH is the referral health facility for Manhiça District, a predominantly rural area with a population of around 200 000 inhabitants^(12)^. The MDH has just over 100 beds including an 8-bed ward dedicated specifically to child malnutrition which has admitted approximately 1500 children each year in the last 5 years (Centro de Investigação em Saúde de Manhiça, unpublished results).

The Centro de Investigação em Saúde da Manhiça (CISM) is a biomedical research centre which has been running a Health and Demographic Surveillance System (HDSS) in Manhiça since 1996^(12)^. According to HDSS data, almost half of the Manhiça population live below the poverty line with subsistence agriculture being the main income activity in the district along with factory work at large sugar factories in neighbouring towns^(13)^. The area is endemic for malaria, tuberculosis, HIV and malnutrition, with hospital-based data collected in Manhiça between 2001 and 2010 showing that 47 % of U-5 patients presented some indication of malnutrition^(13)^. The main staple foods for the district include maize, sweet potatoes, peanuts and cowpea^(13)^. Various nutrition interventions have been implemented in the area over several years, with varying impact/success^(13)^.

### Participants and recruitment

Study participants were recruited at the MDH while attending routine health checks with their U-5’s. As part of these routine health checks, weight, age and gender of the child were recorded on patient health cards. With these data already available, weight-for-age (WfA), an indicator for underweight^(14)^, was used in this study to determine the child’s nutritional status. The child’s weight and age were checked against gender-specific WHO *Z*-score growth charts to determine the child’s WfA *Z*-score. The cut-off points on the growth charts were used to provide an indication of nutritional status by comparing the WfA *Z*-score of the child with the standard reference population^(3)^. Mothers of U-5’s who presented some indication of malnutrition (WfA < –2 sd) and severe malnutrition (WfA < –3 sd)^(14)^ were asked to participate in IDI only, to allow potentially sensitive questions regarding the child’s health and nutrition status to be asked. Mothers of children who did not present any signs of undernutrition (WfA ≥ –2 sd)^(14)^ were asked to take part in either a FGD or IDI. An exception of the FGD inclusion criteria was made for FGD 5 in which one mother of a child with a WfA *Z*-score <–2 sd was included due to low attendance at the MDH that day. No sensitive questions about the child’s weight or health were asked.

Inclusion criteria – FGD: (1) mothers >18 years old with children <5 years old; (2) attending the out-patient paediatric ward and (3) child’s WfA *Z*-score ≥–2 sd of reference population data^(14)^.

Inclusion criteria – IDI: (1) mothers >18 years old with children <5 years old; (2) attending in- or out-patient paediatric wards and (3) children with varying WfA *Z*-scores: ≥–2 sd to <–3 sd of reference population data^(14)^.

Mothers attending the MDH in- and out-patient paediatric wards who met the study inclusion criteria were briefed about the study objective and procedure, that is, a combination of purposive and convenience sampling was applied. Information was given in Changana (the local language) by CISM staff wearing a CISM uniform which mothers visiting the MDH would be familiar with due to the long-standing collaboration between the hospital and the research centre. About 10 % of the mothers who were asked to participate declined, while those (*n* 53) who agreed to participate were provided with informed consent forms which they either read themselves or, for those unable to read/understand, the contents of this form was explained by IN, the second author. Those who signed the informed consent via signature or fingerprint in the presence of a witness were invited to participate in either FGD or IDI.

### Study procedure

Interviews and FGD took place immediately after mothers were recruited at the MDH. The IDI were conducted in Changana and the FGD in a mix of Changana and Portuguese by IN and lasted between 20 and 40 min. The FGD and IDI interview guides (see Annexes) were semi-structured and included questions related to sketched images of infants <6 months with varying mid-upper arm circumference (MUAC) *Z*-scores created for use in another study (see online ‘Supplemental material’, and Table 1 for a description of the images)^(15,16)^. Question topics revolved around mothers’ familiarity with the causes, symptoms and effects of malnutrition, as well as prevention and treatment strategies for malnutrition and what constitutes a sufficient diet for U-5’s. The IDI and FGD were recorded using digital voice recording devices.

**Table 1:** Description of sketched images of infants with varying MUAC *Z*-scores presented to interviewed mothers^(16)^

Image	Nutritional status	MUAC *Z*-score
B	Undernourished	≤–3
P	At risk of malnutrition	>–3 to ≤–2
T	At risk of malnutrition	>–2 to ≤–1
N	Adequately nourished	>–1 to ≤0
K	Well nourished	>0

### Data analysis

Audio records of the interviews were transcribed and simultaneously translated into Portuguese by IN, then translated into English by GP. The interview data were subjected to manifest thematic analysis^(17)^, and dialogical inter-subjectivity was employed as the authors interpreted and compared their findings throughout the analysis. Transcribed and translated material was read several times in Portuguese and English, and during the analysis process, when clarifications were needed, checked by IN against the original recordings.

An abductive approach was taken to analyse the data in which 198 meaning units were identified and descriptively coded. This approach involves an oscillation of the researcher’s thinking between data and theory, that is, between an inductive and deductive approach^(18)^. Therefore, the initial codes generated were data-driven, making the first stage of the analysis inductive; however, after concluding that the initial codes and sub-themes illustrated elements of different levels of health literacy, the second phase of the analysis was theory-driven, thus linking the empirical material with Nutbeam’s discussion of health literacy^(19)^. As this health literacy model is suitable for understanding health-related perceptions and behaviours, it guided the subsequent analysis, rendering the second part of the analysis deductive in character^(20)^. The inductively identified codes were thus grouped in accordance with Nutbeam’s three overarching health literacy themes, then analysed in an iterative process, involving a movement within each theme, between the three themes and the whole dataset. In this last phase of the analysis, nine sub-themes were identified that linked the descriptive codes to the health literacy model (Table 2).

**Table 2:** Results according to sub-theme, theme and link to Nutbeam’s discussion of health literacy

Interpretation of Nutbeam’s health literacy model^(19)^		Interpretation of mothers’ perceptions expressed in interviews		
Level	Definition	Theme	Sub-theme	No of codes
Functional health literacy	Basic factual knowledge on health risks that does not foster skills development or autonomy but enables individuals to act in accordance with instructions.	Functional malnutrition literacy	Varying ability to recognise (un/)healthy physical appearance	62
			Limited ability to identify prevention and treatment strategies	17
Interactive health literacy	A broader range of skills that enable individuals to develop personal capacity to act independently on knowledge, actively seek out health-related information, and increase their motivation and self-confidence.	Interactive malnutrition literacy	References to a biomedical understanding of malnutrition	11
			Fairly developed references to foods appropriate for age	37
			Brief references to allegories or local beliefs	6
Critical health literacy	Knowledge that situates the individuals in a socio-economic context. Includes empowerment as it improves the ability to critically identify options (or the lack thereof), for resilience to social and economic adversity.	Critical malnutrition literacy	Firm requests of information on malnutrition	11
			Basic pursuit of healthcare to reduce health risks	20
			Assertive references to sociocultural conditions	14
			Assertive references to economic conditions	20
			Total	198

## Results and discussion

### Description of study participants

A total of fifty-three mothers participated in the study across nine IDI and seven FGD. Mothers who participated in the study were aged between 18 and 40 years, with the majority of mothers in their late twenties/early thirties. Regarding educational attainment and work situation, the characteristics of the participant group corresponded with those of the population belonging to the MDH’s catchment area, which is reflective of a typical rural district of southern Mozambique^(12)^. Participants had primarily low levels of education (varying between ‘not completed primary education’ and ‘completed secondary education’), and only a few mothers had a formal occupation with most partaking in farming work or were unemployed.

### Themes and sub-themes

Based on the definitions of the three levels of health literacy as proposed by Nutbeam, the descriptive codes and subsequent sub-themes were grouped in relation to ‘Functional Malnutrition Literacy’, ‘Interactive Malnutrition Literacy’ and ‘Critical Malnutrition Literacy’, all of which are presented in Table 2 along with their corresponding sub-themes.

### Functional malnutrition literacy

In total, seventy-nine meaning units were interpreted as mirroring functional health literacy (Table 2), that is, the level that captures factual knowledge which is necessary for uncomplicated, straightforward action.^(19)^ Within these, two sub-themes were identified denoting varying ability to identify healthy appearances and prevention strategies.

#### Varying ability to recognise (un/) healthy physical appearance

Mothers had mixed reactions to the sketched images of infants with varying MUAC *Z*-scores. Some displayed signs of hesitancy, while others confidently described their view of the child in the respective image. Most mothers, however, did not elaborate beyond ‘*healthy*’ and ‘*unhealthy*’. When they did, they mentioned ‘*thin legs*’, ‘*a grown belly*’ and ‘*a head bigger than the body*’ as visible indications of ill health. The more obvious images were correctly identified as presenting healthy (image N and image K) *v*. malnourished children (images P and B). However, image T was largely incorrectly identified as ‘*healthy*’ even though the MUAC *Z*-score (>–2 to ≤–1) indicates the child is at risk of malnutrition.

In addition to the physical attributes, mothers indicated changes in children’s behaviour and appearance as signs of malnourishment:

‘The child with hunger disease does not play like others … It stays isolated, becomes thin, the hair turns brownish. It does not look beautiful’. (FGD 4)

However, mothers acknowledged that they would find it difficult recognising the early signs of malnutrition with one mother saying:

‘When the disease starts you will not see the signs, you will see and watch and when you realise, it may be there already and it is when you are going to take it to the hospital’. (FGD 4)

The lack of profundity in the mothers’ discussions surrounding early sings of malnutrition warrants further research into identifying the point at which mothers/caregivers recognise the risk or presence of malnutrition in children, as well as the point that triggers concern and action on the part of caregivers. Descriptions of the symptoms of malnutrition resembled that of severe protein-energy malnutrition manifested as marasmus or kwashiorkor. This finding is consistent with previous literature in which children with comparable symptoms were recognised as being unhealthy, whereas short stature was considered the norm in communities with a high prevalence of linear growth faltering^(5,6,9,16,21,22)^. This indicates a potential lack of awareness of other forms of malnutrition, specifically stunting. Further evidence providing a basis for context-relevant awareness-raising campaigns informing about all forms of malnutrition seem desirable, especially since the most common forms of malnutrition in Mozambique include stunting and Fe deficiency anaemia^(2)^.

#### Limited ability to identify prevention and treatment strategies

The meaning units gathered under this sub-theme displayed a limited malnutrition literacy. Attending the hospital for routine weighing was the only prevention measure mentioned. Monitoring the weight of the child and following instructions from hospital staff was also highlighted as a key treatment method. In general, the mothers’ responses exhibit low self-efficacy in preventing and treating malnutrition saying that they simply ‘*don’t know*’ how they would treat or prevent malnutrition and that they would have to seek advice from the hospital or ‘*other people*’. Their inputs into the discussion of prevention and treatment in this interview format were thus poor.

### Interactive malnutrition literacy

This level of health literacy captures the kind of knowledge that allows the individual to evaluate different forms of information and confidently make independent health-related decisions. We identified fifty-four meaning units, abstracted into three sub-themes that illustrated this kind of literacy. When mothers conceptualised the causes and effects of ‘*hunger disease*’, they primarily formulated this in terms of biomedical understandings, foods for age and local allegories or beliefs related to parenting.

#### References to a biomedical understanding of malnutrition

Mothers’ descriptions of the causes of malnutrition were predominantly in line with biomedical concepts (lack of food, care and hygiene). Some demonstrated a basic awareness of the comorbidities of malnutrition (diarrhoea, tuberculosis, HIV/AIDs). However, the data also suggest that malnutrition is sometimes misdiagnosed within the community:

‘In the community whenever the baby gets sick, even if you’re an adult, they say it’s HIV … they don’t say anything else in the community’. (FGD 5)

While most talk revolved around acute malnutrition, there were also a couple of references made to chronic malnutrition, for example, to pre- and postnatal factors:

‘If the mother did not feed well, of course the baby can be born with low weight’ (FGD 7).

The analysis shows an ability to reason and evaluate different biomedical explanatory models (causes and comorbidities) of malnutrition; however, the interviews did not give sufficient information for evaluating whether it was used as a base for nutritional strategies.

#### Fairly developed references to foods appropriate for age

Mothers displayed a higher level of awareness of the link between food and malnutrition. Inaccuracy in assessing a child’s need for food as well as an absence of ‘*good food*’ were identified as causes of malnutrition. However, perceptions often seemed to be based on texture rather than nutritional quality with ‘*light*’ foods considered suitable and ‘*heavy*’ foods unsuitable for U-5’s. When listing foods considered good for growth and development, xima (a thick porridge made from maize or corn flour), soups and curries enriched with peanuts were frequently mentioned. Cold foods were considered harmful to the child’s ‘*organism*’ resulting in an upset stomach.

Above all, mothers emphasised the importance of age-appropriate foods, for example, to not give juice and biscuits as ‘*it is not the essentials that children need … at the age they have*’ (FGD 7). A distinction was also made between foods appropriate for adults and children but mothers explained that because of a lack of *‘suitable’* food, adult food is sometimes given.

‘This ends up causing this malnutrition, because … the baby’s organism can hardly digest those foods that an adult consumes’. (FGD 5)

Meat and animal-source foods were noticeably absent in mothers’ responses, while ‘*rich food*’ described as vegetables and enriched porridges were recognised as foods that could be used to treat malnutrition. It is not clear from the data whether animal-source foods are purposively avoided due to food taboos or are considered inaccessible due to financial or structural constraints as identified in a previous study conducted in northern Mozambique^(23)^. Considering the nutritional importance of animal-source foods for infants and children in providing high-quality protein and a range of key micronutrients conducive to the healthy growth and development of U-5’s^(24,25)^, further research into the root cause of this finding is needed.

The mothers’ conceptualisation of the link between malnutrition and food perceived to be appropriate for U-5’s was detailed with varied examples and strategic choices described in a more elaborate and confident manner compared to the other sub-themes.

#### Brief references to local allegories or beliefs

When mothers were asked how malnutrition is perceived in their community, the expression *djambelada* [the child ‘had been jumped’] was mentioned:

‘They say “that child was djambelada”. It has lean legs, the belly grew and the head. They say that it is a problem of hunger disease … You cannot djambelar while you are still breastfeeding’. (FGD4)

The literal meaning of this expression is that the mother, instead of letting the child sleep between the parents, had ‘jumped’ over the child and slept with her husband. This expression was also used with reference to breast-feeding and hygiene:

‘When you like to sleep with the father while the baby is still small, and when you have done that you touch the child without having showered or washed your hands and you breastfeed the baby, that’s bad for the child’. (FGD 6)

It is, from our material, not obvious to what extent the expression is an allegory for the mother not making the child’s needs a top priority or whether this is an explanatory model locally accepted as a cause of malnutrition. Regardless, the analysis indicates a general use and awareness of the expression. However, the way the mothers discussed this indicates that they perceived themselves as being aware of a variety of knowledge regimens related to behaviours and their link to the child’s well-being. Since previous research has acknowledged the great significance of incorporating local beliefs into health intervention programmes to increase their efficacy^(9)^, gaining a better understanding of the significance of these local beliefs can allow a more comprehensive decision to be made about how they could be integrated into future interventions.

### Critical malnutrition literacy

The remaining sixty-five meaning units, structured into four sub-themes, were interpreted as being linked to the third and most advanced form of health literacy as proposed by Nutbeam. This level captures the connection between health-related knowledge and the broader societal context, that is, the kind of knowledge that is needed to act gainfully in a specific sociopolitical context^(19)^. However, contrasting the Nutbeam model, this part of the material did not primarily display empowerment through knowledge to address social and economic determinants to improve health, but rather that the mothers perceived themselves at the losing end of inequalities^(19)^.

#### Firm requests for opportunities to learn about malnutrition

Mothers expressed a desire to learn more about malnutrition by directly requesting more information, for rexample, from hospital staff about general care practices, appropriate foods for children, causes of diarrhoea and causes of malnutrition:

‘A small lecture about what we can change in the diet … Teach us to do these things without needing a lot of appliances at home … to improve the condition of children’. (FGD 7)

The interviewees clearly expressed awareness of the need to increase their knowledge of malnutrition as well as an eagerness to improve their parental strategies to promote their children’s nutrition and health. Therefore, the results imply that future campaigns which aim to increase malnutrition literacy among caregivers would be welcomed in this setting.

#### Basic pursuit of healthcare to reduce health risks

This sub-theme is obvious throughout the dataset with mothers indicating that they would seek medical help at the hospital upon identifying physical or behavioural changes in their child such as weight loss, loss of appetite or a reluctance to be breastfed:

‘If I stayed at home I wouldn’t know how to deal with this…. There is no other alternative, when the child is sick we go to the hospital and there they will know what to do. We cannot do anything else but that’. (FGD 1)

Mothers also reported recommending other mothers/caregivers to seek medical help to reduce health risks and emphasised the importance of following the instructions given at the hospital on how to rehabilitate the child. Importance was put on attending the hospital for an accurate diagnosis, since mothers alone may not be able to correctly identify what illness the child has:

‘I can see a child with nothing, even though it has anaemia. You think she has a good body, but it is sick’. (IDI 6)

While mothers made references to seeking care at the hospital upon noticing signs of ill health, the identification of signs differed as well as mothers’ timing of seeking medical help. One depicted determinant of this was the proximity of the hospital from the home, with further distance and the need for money for public transport resulting in a delay in seeking treatment.

‘If you live far away from the hospital you may wait longer before you go, because to go to the hospital you need money for the bus. Those who live far away know that, but if you are close you can walk to the hospital’. (FGD 5)

The many references to the district hospital were most likely influenced by the study location being the hospital. Nevertheless, it is still noteworthy that there was a reliance and usage of the hospital in identifying and treating malnutrition, since similar studies conducted in community settings indicated a greater reliance on traditional doctors, religious leaders and community members to treat children with symptoms of malnutrition^(5,6,9)^.

While the findings show that mothers value the services of the MDH, a delay in seeking treatment may be the consequence of: (1) structural barriers such as proximity to the MDH; (2) economic barriers including the cost of transport to the MDH and (3) potential health literacy barriers as the interviewed mothers had difficulties in identifying the early stages of undernutrition. Therefore, even among service users of the MDH, the culmination of these barriers may result in malnutrition progressing to severe stages before the child reaches the hospital. Investigations into how different institutions can be strengthened to support mothers in preventing, detecting and treating malnutrition are therefore recommended, including investigations into what different roles general poverty reduction strategies, community members and local healthcare facilities can play for preventive strategies, early detection and treatment of late stages of malnutrition.

#### Assertive references to sociocultural conditions

Quality of parenting, parental educational level and attitudes towards feeding were, according to some mothers, related to malnutrition. In these general discussions, the term *Djambelar* was not used. Mothers emphasised the importance of paying attention to children, citing ‘*lack of attendance to the child*’ (FGD 1) as a cause of malnutrition and ill health. Mothers returning to work soon after giving birth and leaving the child in the care of someone else, particularly while the child is still of breast-feeding age, and the subsequent pre-mature weaning and increased risk of malnutrition that results from this^(26)^, was mentioned as a cause of malnutrition:

‘Sometimes we leave children with other people … who don’t give food the way it should be, or the mother has left a child that is breastfed and someone else is making milk and they do not follow the instructions. This can be bad for the child’. (FGD 5)

Due to work-related time constraints, mothers reported opting for convenience foods over home-cooked foods despite being aware that this is not ideal. This growing trend of preferences towards ready-to-eat convenience foods was identified as a factor associated with malnutrition.

‘Some mothers work already and do not have time and prefer to buy. Home-grown food is more important than that food you can buy’. (IDI 1)

Household food insecurity due to the presence of many family members was also identified as a cause of malnutrition. Some linked household food insecurity to the burden of feeding grandparents, yet others mentioned them as a source of knowledge and support, and indicated that the shift in parenting between generations, towards preferences for more convenient foods was a contributor to malnutrition.

#### Assertive references to economic conditions

There was a strong awareness of the link between poverty and malnutrition with mothers stating that they sometimes do not have the financial capacity to provide foods for their children never mind ‘*good foods*’.

‘You also have other mothers who want to give, but do not have the means to give every day, not even a little bit of food’. (FGD 7)

A lack of financial support, possibly stemming from extreme poverty and resulting in chronic food insecurity, was a reoccurring theme.

‘There is nothing in place to help when the child is born, you do not know what to do. Even the mother does not have good food to eat, she has milk to breastfeed the child but she does not have the conditions that could help her give other things to the child, so the child grows in suffering from the start’. (IDI 5)

Lack of income, with subsequent food insecurity, was similarly mentioned as a cause of malnutrition:

‘I sometimes have difficulties, when I do not work and the father of the child also does not work and we stay at home, it is difficult to guarantee good nutrition for the baby. Those who have a job, they always manage to get something for their baby… There are mothers who do not manage because life is not easy’. (FGD 6)

While mothers linked poverty directly with a lack of food, it was also linked with a lack of other resources that impact a child’s general health:

‘Our reality dictates what we do, for example I live in an area that has no energy… When there is no freezer this [breast] milk will be ruined after two hours … so it is impossible to take milk from the breast … I have to resort to the cans’. (FGD 7)

‘If there is no way to change, for example be born by another mother, sometimes the only thing you can do is to regret being born. What will you do if there is no one to help you? No brother, born lonely, then it is difficult to find the money’. (IDI 5)

This sub-theme presents mothers’ awareness of not only the different links between the causes of poverty and malnutrition, but also the debilitating role that poverty plays in dictating caregivers’ ability to change their situation and respond appropriately to health challenges. These findings are consistent with similar studies which also found poverty to be a perceived cause of malnutrition^(10,21,27)^. While mothers linked malnutrition directly to poverty and a lack of resources, references to economic barriers in some findings were implicit, for example, mothers’ focus on providing enough calories as opposed to a variety of foods could be the consequence of systemic poverty, rather than simply a lack of knowledge at the individual level on what constitutes a sufficient diet for children^(4)^. This is consistent with the findings presented in the Government’s Multisectoral Action Plan for the Reduction of Chronic Undernutrition 2011–2015, which states that poorly diversified, low nutritional quality diets due to poverty and household food insecurity contribute to the high prevalence rates of stunting, anaemia and other micronutrient deficiencies in Mozambique^(4)^.

### Strengths and limitations

The exploratory nature of the study implies that indications for future research, rather than thematic, theoretic or data saturation, was sought. This, together with the fact that the choice of study site may have caused a sampling and response bias, should be taken into consideration, and findings should therefore be viewed as preliminary and indications of topics for further research. The use of images of children with varying MUAC measurements to guide the discussions and WfA as a recruitment measure potentially resulted in a biased response with the focus of the discussions of malnutrition on underweight and wasting, while stunting went virtually unmentioned. The abductive approach strengthened the study’s validity by making possible triangulation of the empirical material through the connection to a broader body of scientific work^(17,18,28)^. Conducting the interviews in the local language is considered a strength as well as the research team’s local expertise and broad competency in areas, including nutrition, anthropology, public health and medicine.

## Conclusions

The key findings, which emerged from this research, were that the perceived symptoms of malnutrition largely related to protein-energy malnutrition with symptoms of stunting and micronutrient deficiencies went largely unmentioned. Mothers’ awareness of the multifaceted causes of malnutrition was more developed than their perceived ability to identify and treat malnutrition. This, together with mothers’ willingness to increase their nutrition literacy through training by health professionals, shows the need and want for health literacy promotion programmes.

The reliance of the MDH medical professionals to identify and treat malnutrition in this community and the lack of references to community health workers highlights the need to explore the possibility for community health posts with greater proximity to rural populations, to play a greater role in early identification and prevention of malnutrition in U-5’s. The limited ability of mothers in identifying malnutrition prevention and treatment strategies emphasises the need to enhance caregivers’ nutrition health literacy, integrating local conceptions and contextual factors, such as food norms and income level, when developing programmes to reduce malnutrition rates. Finally, the prominence of poverty and sociocultural conditions in mothers’ responses as links to malnutrition identifies areas for in-depth research, preferably with a focus on how malnutrition prevention efforts can consider how mothers’ actual capacity to promote their children’s nutritional well-being is linked to their financial situation and degree of institutional and structural support.

Further research, which aims to gain a full understanding of mothers’ knowledge of the different forms of malnutrition, is paramount in guiding health promotion programmes focused on nutrition. Investigations into social policies, food accessibility and availability, local beliefs and food taboos present in this context seem necessary to make a more comprehensive decision on how these influencing factors can be incorporated into existing and future nutrition interventions to improve their efficacy. Therefore, community-based participatory research that seeks to (1) uncover the knowledge required by caregivers on malnutrition, taking socio-economic conditions into account, to increase nutritional literacy and self-efficacy in treating malnutrition and (2) identify the forms in which this information should take in order to be communicated and understood effectively is recommended to increase the chances of success at reducing the high prevalence of malnutrition in U-5’s.

## Supplementary material

For supplementary material accompanying this paper visit https://doi.org/10.1017/S1368980021004365. click here to view supplementary material
