# Supplementary material for: A qualitative study of mothers’ health literacy related to malnutrition in under 5-year-old children in southern Mozambique
Source: Public Health Nutr. 2021 Nov 15;25(7):1947–55. doi: 10.1017/S1368980021004365 (PMC9991675; doi:10.1017/S1368980021004365)
Supplement: Supplementary file 1 [file S1368980021004365sup001.docx]

## Annex 1: Focus group discussion interview guide Manuscript ID PHN-RES-2021-0668

**Focus group discussions interview guide**

Intro question: How was your hospital visit today?

*Discussion questions*

*Part 1: Pictorial related questions*

1. What do you see when you look at these pictures? *[Probe: Do these children have any problems or illnesses? Can you describe what these problems or illnesses are? What are the signs that make you think the child has a problem/illness?]* Do you have any ideas around what could cause these problems/illnesses? Can you explain why these things cause the illness?
2. Have you heard the term malnutrition before? Can you describe what it means, what it is?
3. Do you think any of these children have malnutrition? (Pictorials) [*Probe: Which ones? How do you know? What are the signs showing malnutrition?]*
4. Why do you think children come to be malnourished? [*Probe: what do you think causes this?]*
5. When someone in this community sees a child with malnutrition, what do you think that they think about it?
6. If your child has malnutrition what do you think you would do to help them get better and why? [Probe: *Can you explain a little bit more around this choice? What is it that you think would help your child to get better?]*
7. After seeing your child is malnourished, how long do you wait before you do something to help them get better?
8. Do you think there are some bad things that can happen to a child’s health if they have malnutrition? [*Probe: What will happen? Will they get sick soon after? How will they get sick? Will they get* sick in the future? *How will they be sick in the future?]*

*Part 2: Questions about the diet of under 5s*

1. For those with children above 6 months, what does your child eat in a typical day? Why these foods?
2. What do you think about breastmilk and breastfeeding? How long is it good to breastfeed your baby for? [*Probe: How many weeks/months/years?]*
3. Can you describe the best foods for a child that is a little bit older, when they can sit up, start walking but cannot talk yet? How many times per day would you feed a child this age?
4. Can you describe what are the best foods when a child is even bigger, when they can walk and talk and start to play with other children? How many times per day would you feed a child this age?
5. Do you think some people can’t or don’t give these foods to their child? [*Probe: Why do you think this is?]*
6. Is there anything else you want to add about what we discussed?

*Close*

Thank you for your time and valuable insights. As mentioned in the consent form, all the information taken from this discussion will be completely anonymous.

## Annex 2: Individual interview guide Manuscript ID PHN-RES-2021-0668

**Individual interview guide**

*Opening*

Thank you for volunteering your time to take part in this interview. As mentioned, this interview shouldn’t take longer than 40 minutes. Do you have any questions about the consent form? We will begin by collecting your signed consent form.

Close-ended questions;

1. How old are you?
2. How old is your child? (the one attending the hospital/admitted)
3. How many children have you given birth to?
4. Have you spoken to any of the nurses before about feeding your baby?

Open-ended questions

1. What do you see when you look at these pictures? [Probe: *Do these children have any problems or illnesses? What are these problems or illnesses?]*
2. Have you heard the term malnutrition before? Can you describe what malnutrition means, what it is?
3. Do any of these children have malnutrition? [*Probe: which ones? How can you tell?]*
4. How do you know that a child has malnutrition [*Probe: Can you see that when you look at the child or how do you know?]*
5. Why do children come to be malnourished? [*Probe: In your opinion what causes malnutrition?]*
6. How do you prevent a child from becoming malnourished?
7. When people in your community see a child with malnutrition, what do you think they think about it?
8. If your child had malnutrition (or wasn’t eating enough food) what do you think you would do to help him/her get better?
9. After seeing your child with malnutrition (or losing weight) how long do you wait before you do something to help them get better? Why this amount of time?

*Part 2: Questions about the diet of under 5s*

1. What does your child eat in a typical day? [*Probe: what kinds of foods?]* Why these foods?
2. How many times a day does your child eat?
3. What are your views on breastmilk and breastfeeding? How long is it good to breastfeed your baby? [*Probe: how many weeks, months, years]*
4. What to you are the best foods for a child to eat so that the child can grow to be big and strong?
5. Why do you think some mothers don’t give these foods to their child? *[Probe: What stops mothers giving these foods to their child?]*
6. Is there anything else you want to add about what we discussed?

*Close*

Thank you for your time and valuable insights. As mentioned in the consent form, all the information taken from this discussion will be completely anonymous.
